# Characteristic Polyphenols in 15 Varieties of Chinese Jujubes Based on Metabolomics

**DOI:** 10.3390/metabo14120661

**Published:** 2024-11-28

**Authors:** Yong Shao, Siying Li, Xuan Chen, Jiahui Zhang, Huxitaer Jianaerbieke, Gang Chen, Xiaodong Wang, Jianxin Song

**Affiliations:** 1 Key Laboratory of Geriatric Nutrition and Health, Ministry of Education, Beijing Technology and Business University, Beijing 100048, China; luckystarsy@163.com (Y.S.); gang.chen@btbu.edu.cn (G.C.); 2 School of Functional Food and Wine, Shenyang Pharmaceutical University, Shenyang 110016, China; milu2175@outlook.com (S.L.); 2439777451@163.com (X.C.); 1372774626@163.com (J.Z.); 1726505908@163.com (H.J.); 3 School of Biological Science and Food Engineering, Chuzhou University, Chuzhou 239000, China; wangcy451@163.com

**Keywords:** ripe dried jujubes, polyphenol, variety, metabolomics, PCA

## Abstract

Background: Jujube is a homologous herb of medicine and food, and polyphenols are key in determining the functional effects of jujubes. Methods: In this study, characteristic polyphenols in 15 varieties of Chinese jujubes were investigated based on untargeted metabolomics. Results: The results showed that a total of 79 characteristic polyphenols were identified in the 15 varieties of Chinese jujube, and 55 characteristic polyphenols such as syringetin, spinosin and kaempferol were reported for the first time. Scopoletin (63.94% in LZYZ), pectolinarin (22.63% in HZ) and taxifolin (19.69% in HZ) contributed greatly and presented significant (*p* < 0.05) differences in the 15 varieties of Chinese jujubes. HZ was characterized by pectolinarin, erianin and wogonoside, while XSHZ, NYDZ and RQHZ, with similar polyphenol profiles, were characterized by (+)-catechin, combretastatin A4 and tectorigenin. JSBZ, HMDZ, TZ, JCJZ and HPZ had similar polyphenol profiles of galangin, isoferulic acid and hydroxysafflor yellow A. Conclusions: Metabolomics is critical in grasping the full polyphenol contents of jujubes, and the differences in the polyphenol profiles and characteristic individual polyphenols of the 15 varieties of Chinese jujubes were well analyzed by principal component analysis (PCA).

## 1. Introduction

Jujube (*Ziziphus jujuba* Mill.), also called red jujube or Chinese date, belongs to the Rhamnaceous family and is widely cultivated and consumed worldwide [1]. Jujube originates from China, with a history of 7000 years, and more than 400 cultivars are available [2,3]. In Chinese traditional culture, and according to the latest announcement by the National Health Commission and State Administration for Market Regulation of China (2024), jujube is a homologous herb of food and medicine. Research has shown that jujube is rich in carbohydrate, proteins, amino acids, minerals and various bioactive compounds such as polyphenols, flavonoids, saponins and polysaccharides [4,5]. Therefore, the bioactive activities, such as its antioxidant, anticancer and anti-insomnia activities, of jujubes are excellent [6]. Jujubes are classified as dried, candied, multipurpose and ornamental, and dried ones account for over 80% of all jujubes [7,8]. In daily life, jujubes are eaten fresh and dried directly or consumed as jujube wine, compotes, jujube juice, bread, cake and yogurt [1,3].

As a kind of secondary metabolite, polyphenols widely exist in plants and plant-based foods, and they can be classified into flavonoids, non-flavonoids and tannins based on their characteristic chemical structure of aromatic rings with one or more hydroxyl moieties [9,10]. Polyphenols present a variety of functional effects such as antioxidative and antiglycative activities and antimicrobial, antiproliferative and anti-inflammatory properties and are closely related to the water-holding capacity, texture and color of foods [11,12]. Wang et al. (2022) [13] found that the total content of phenols and flavonoids was 1.33~10.92 mg/g and 0.10~7.66 mg/g, respectively, in Chinese jujubes. According to current statistics, over 60 polyphenols have been reported in jujubes, and caffeic acid, rutin, protocatechuic acid, ferulic acid, gallic acid, *p*-coumaric acid, quercetin, coumarin and (-)-epicatechin are common [14,15,16,17]. The characteristic phenolics in jujubes are significantly affected by maturity stages [15,16], processing conditions [18] and varieties [13,19]. It is noteworthy that a total of 59 individual phenolic compounds have been reported in jujubes by metabolomics [14]. However, only seven phenolics have been detected in 26 varieties of jujubes by LC/MS [13], which indicates that metabolomics is superior in phenolics analysis.

Metabolome analysis has the advantage of identifying the whole metabolites (<1000 Da) in plants, foods and humans [20,21]. In practical applications, gas chromatography–mass spectrometry (GC-MS), liquid chromatography–mass spectrometry (LC-MS) or nuclear magnetic resonance (NMR) is applied for metabolome characterization [22,23]. Unlike GC-MS, adapted for the detection of low-molecular-weight metabolites [24,25], metabolome characterization based on LC-MS can achieve the best metabolite coverage, covering everything from very polar to non-polar food molecules [26]. Recently, metabolome analysis based on LC-MS has been successfully applied for the characteristic polyphenol analysis of strawberry [27], green tea [28] and barley [29]. Hence, in order to reveal the characteristic polyphenol compositions in commercial Chinese jujubes, metabolomics analysis based on LC-MS was applied in this study. Moreover, the differences in 15 commercial Chinese jujubes were distinguished based on their polyphenols as well.

## 2. Materials and Methods

### 2.1. Plant Material

**Figure 1:**
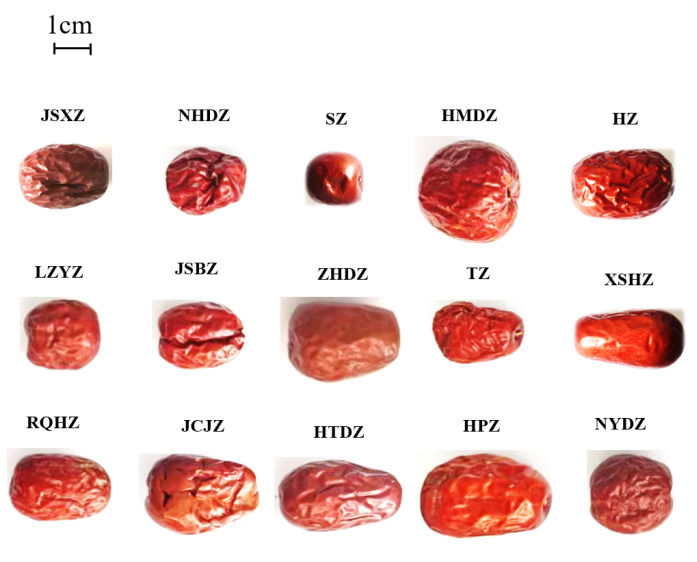
Pictures of 15 varieties of Chinese jujubes.

A total of 15 commercial Chinese ripe dried jujube fruits were collected from markets in different regions of China. These jujubes were as follows: JSXZ (Jin Si Xiao Zao, originating from Cangzhou, China), NHDZ (Nei Huang Da Zao, originating from Anyang, China), SZ (Suan Zao, originating from Kashi, China), HMDZ (Ha Mi Da Zao, originating from Hami, China), HZ (Hui Zao, originating from Akesu, China), LZYZ (Lin Ze Yu Zao, originating from Zhangye, China), JSBZ (Ji Shan Ban Zao, originating from Jishan, China), ZHDZ (Zan Huang Da Zao, originating from Zanhuang, China), TZ (Tan Zao, originating from Jinzhong, China), XSHZ (Xi Sha Hong Zao, originating from Zhongning, China), RQHZ (Ruo Qiang Hui Zao, originating from Ruoqiang, China), JCJZ (Jiao Cheng Jun Zao, originating from Jiaocheng, China), HTDZ (He Tian Da Zao, originating from Hetian, China), HPZ (Hu Ping Zao, originating from Jinzhong, China) and NYDZ (Ning Yang Da Zao, originating from Ningyang, China). About 2.0 kg of each variety of commercial Chinese jujubes was obtained and stored at 4 °C in packaging. Pictures of the 15 varieties of commercial Chinese jujubes are shown in Figure 1.

### 2.2. Metabolite Extraction

The metabolite extraction of the 15 varieties of commercial Chinese jujubes was conducted according to the method of Song and Tang (2023), which was optimized by Novogene Co., Ltd. (Beijing, China), and it was efficient and accurate [22]. Briefly, accurate samples of 100.00 mg of jujube (ground with liquid nitrogen) were, respectively, put in PE tubes. Then, 500 μL of 80% aqueous methanol solution containing 0.1% formic acid (LC-MS grade) was added and treated with vortex concussion. Subsequently, the samples were kept in an ice bath (5 min) and then centrifugated at 15,000× *g* at 4 °C (10 min). The resulting supernatant was diluted to a 53% methanol content. Eventually, the supernatant was collected for injection and analysis.

### 2.3. LC-MS/MS Analysis

A Vanquish UHPLC (Thermo Fisher, Bremen, Germany) coupled with a Q ExactiveTM HF mass spectrometer (Thermo Fisher, Germany) was used for the UHPLC-MS/MS analysis, and a C^18^ column (Hypesil Gold, 100 × 2.1 mm, 1.9 μm, Thermo Fisher, Lenexa, KS, USA) was equipped. LC-MS-grade formic acid (0.1%) and methanol were used as mobile phases A and B, respectively, for the positive-mode analysis, and ammonium acetate (5 mM, pH 9.0) and methanol were used as mobile phases A and B, respectively, for the negative-mode analysis. The column temperature and the flow rate were 40 °C and 0.2 mL/min, respectively. The gradient elution was performed as follows: 2% B, 1.5 min; 2–85% B, 3 min; 85–100% B, 10 min; 100–2% B, 10.1 min; and 2% B, 12 min. The Q ExactiveTM HF mass spectrometer was operated in the positive/negative polarity mode (3.2 kV spray voltage, 320 °C capillary, 40 arb sheath gas and 10 arb aux gas).

### 2.4. Metabolite Identification

Peak alignment, peak picking and quantitation of each metabolite was conducted using the Compound Discoverer 3.1 (CD3.1, Thermo Fisher) of UHPLC-MS/MS device. The tolerance values of the retention time, actual mass and signal intensity were 0.2 min, 5 ppm and 30%, respectively, while the signal/noise ratio and minimum intensity were 3 and 100,000, respectively. All the peak intensities were normalized to the total spectral intensity. Based on the additive ions, molecular ion peaks and fragment ions, the normalized molecular formulas were predicted. Then, the identified polyphenols that presented at least a full match with the mzCloud (https://www.mzcloud.org/, accessed on 24 June 2024), mzVault or MassList databases were analyzed [30]. R (R version R-3.4.3) was used for the statistical treatment. All determinations were analyzed by Novogene Co., Ltd. (Beijing, China).

### 2.5. Data Preprocessing

The area normalization method was used for the content analysis of each identified polyphenol [31]. SPSS version 20.0 (SPSS Inc., Chicago, IL, USA) was used for verifying the difference analysis by Duncan’s multiple tests at the *p* < 0.05 level. All determinations were repeated three times, and the results are presented as mean ± SD (standard deviation) [8]. Principal component analysis (PCA) was carried out using the EZinfo 3.0 software. The average value was selected for PCA, and the total of two principal components was over 80%, which was enough to explain the dataset.

## 3. Results and Discussion

### 3.1. Identification of Characteristic Polyphenols in 15 Chinese Jujubes

The characteristic polyphenols in the 15 Chinese jujubes were identified based on untargeted metabolomics, and they are listed according to their retention time (RT) in ascending order. Information regarding the compound, m/z, CAS number, molecular weight, molecular formula and molecular structure of each individual polyphenol is presented as well (Table S1), and the identified compositions that showed at least a full match with the mzCloud, mzVault or massList databases in the UHPLC-MS/MS results were analyzed.

Table S2 shows a total of 79 characteristic polyphenols, which were identified in the 15 varieties of Chinese jujube. Among these polyphenols, rutin, ferulic acid, (+)-catechin, *p*-coumaric acid, (-)-epicatechin and quercetin were common in the jujubes [13,15,17,32,33]. However, in previous studies, only 7 polyphenols were reported in 26 varieties of Chinese jujubes [13], 10 polyphenols were determined in ‘Junzao’ jujubes [32], 12 polyphenols were found in 7 cultivars of Chinese jujube [17], 10 polyphenols were analyzed in 7 varieties of Chinese jujubes [15] and 8 polyphenols were presented in Xinjiang jujubes [33], according to high-performance liquid chromatography (HPLC) analysis. This indicates that the metabolomics method in this study was superior in terms of analyzing the polyphenols of jujube, which agrees with the result of Zhang et al. (2023) [14], where 59 phenolics were detected. Moreover, phloretin, resveratrol, trilobatin, taxifolin, nobiletin, scopoletin, eriodictyol, myricetin, isorhamnetin, luteolin and naringenin, which were detected in this study, were also analyzed previously [14]. Importantly, a total of 55 characteristic polyphenols, such as syringetin, spinosin, kaempferol and apigenin, were found in jujube for the first time [1,13,14,15,17,32,33], which is critical in understanding the full nutritional range of components in jujube and then deeply exploring the functional effects of jujubes.

### 3.2. Content of Characteristic Polyphenols in 15 Chinese Jujubes

In this study, the area normalization method was applied for the content analysis of the characteristic polyphenols in 15 Chinese jujubes, and the individual phenolics with a content of >1% are shown in Table 1. With a significantly high content, scopoletin (63.94% in LZYZ), pectolinarin (22.63% in HZ), taxifolin (19.69% in HZ), camelliaside A (18.82% in NYDZ), combretastatin A4 (16.25% in XSHZ) and *p*-coumaric acid (10.67% in JSXZ) contributed greatly to the total polyphenol content of the different varieties of jujube (Table 1). Scopoletin, which acts as a derivative compound of coumarin and is widely present in noni, is closely related to the antioxidative, immunomodulatory, anti-inflammatory and hepatoprotective properties that determine the functions of jujube [34]. Taxifolin is a kind of dihydroflavonol and presents strong antiviral, anti-aging, anticancer, anti-inflammatory and antiangiogenic effects, as well as contributing to immunity regulation and liver protection [35]. *p*-Coumaric acid shows antioxidant, anti-inflammatory, anti-apoptotic, neuroprotective and memory-ameliorating effects, and it can also promote hippocampal neurogenesis and stimulate hippocampal synaptic plasticity and protect against scopolamine-induced hippocampal LTP degradation and cognitive impairment [36]. However, significantly different contents of scopoletin (from 0.03% to 63.94%), pectolinarin (from 0.46% to 22.63%), taxifolin (from 0,15% to 19.69%), camelliaside A (from 0.02% to 18.82%), combretastatin A4 (from 0.06% to 16.25%) and *p*-coumaric acid (from 0.31% to 10.67% in JSXZ) were, respectively, present in the 15 varieties of Chinese jujubes, indicating the different bioactive abilities of the 15 varieties of Chinese jujubes.

Ferulic acid, (+)-catechin, epicatechin and coumarin are common in jujubes, and they presented significant (*p* < 0.05) differences in the 15 varieties of Chinese jujubes, with contents of 0.18% (in HZ)−4.31% (in JSXZ), 0.02% (in SZ, LZYZ and JSBZ)−1.08% (in XSHZ), 0.12% (in SZ)−5.49% (in RQHZ), and 0.08% (in HZ)−3.00% (in HTDZ), respectively (Table 1). Similar results were also reported in an analysis of the phenolic compounds of 26 varieties of jujubes [13]. Ferulic acid has the ability to reduce body weight, modulate the gut microbiota composition in high-fat diet (HFD)-induced mice and improve glucose and lipid metabolisms by activating the insulin receptor/phosphatidylinositol 3-kinase/protein kinase B (PI3K/AKT) pathway [37]. (+)-Catechin is a potent antioxidant with therapeutic benefits against cancers, coronary heart disease and inflammatory disorders [38]. Epicatechin is excellent in terms of antioxidation and anti-inflammatory activity and in increasing the activity or expression of antioxidant enzymes [39]. Other polyphenols, such as syringetin, erianin, wogonoside and galangin, which were found here for the first time, also contributed to the characteristic polyphenol profile of the jujubes (Table 1). This indicates that the biological activities of different varieties of jujubes are significant due to their different characteristic phenolics content. Furthermore, metabolomics provides technical support towards discovering the total nutrient content of jujubes and exploring their functional effects.

### 3.3. PCA of 15 Commercial Chinese Jujubes

**Figure 2:**
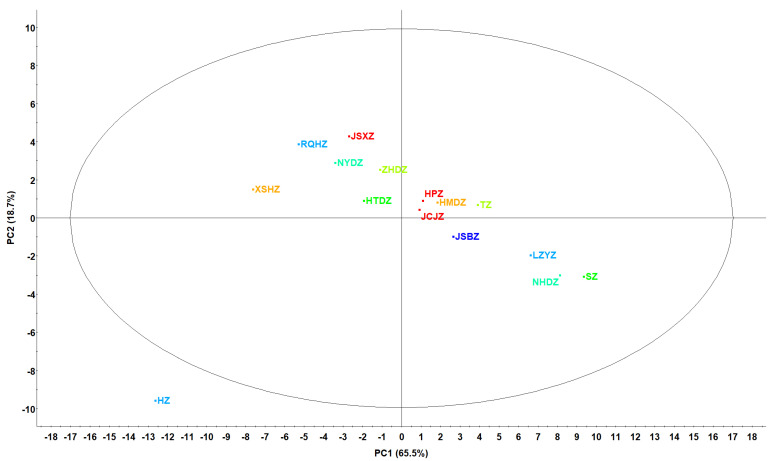
PCA of 15 commercial Chinese jujubes based on their whole polyphenol contents.

As a multivariate statistical method, principal component analysis (PCA) has the advantages of analyzing complex datasets by identifying two or more principal component factors and examining the correlations between multiple variables [4,40]. To accurately distinguish the differences in the polyphenol profiles and determine the characteristic individual polyphenol profiles of the different commercial Chinese jujubes, PCA was applied according to the content of the identified polyphenols (Table S1 and Table 1). As shown in Figure 2, a 2D PCA plot was established with the total contents of polyphenol compounds (Table S1), and the total contribution of PC1 (65.5%) and PC2 (18.7%) was 84.2%, which is enough to explain the dataset [24]. As shown in the PCA plot (Figure 2), the observations of the 15 commercial Chinese jujubes were well separated, indicating that the differences in their polyphenol profiles could be accurately analyzed by PCA. HPZ, HMDZ, JCJZ, TZ, JSBZ and HTDZ showed close distances to each other, indicating they have similar polyphenol profiles. Moreover, HZ presented a large distance from the other jujube samples, and SZ (NHDZ, LZYZ) was also far away from XSHZ (RQHZ, JSXZ), indicating they have different polyphenol profiles, respectively [41].

**Figure 3:**
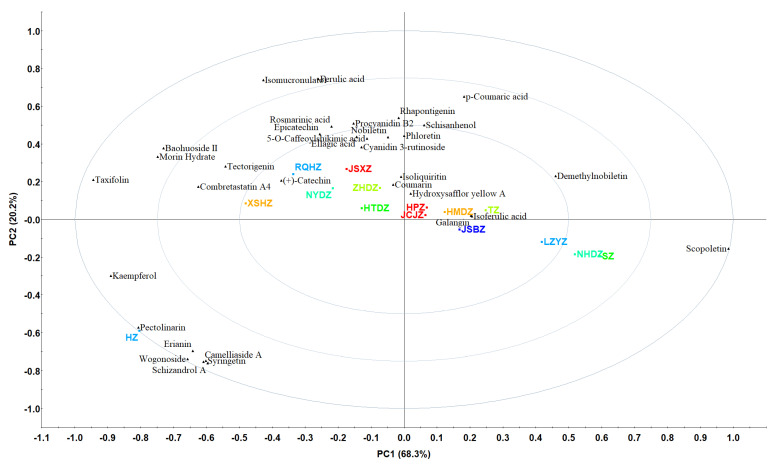
PCA of 15 commercial Chinese jujubes based on the content of polyphenols > 1%.

To clarify the critical and characteristic individual polyphenol contents of the different varieties of jujubes, the characteristic polyphenols in the 15 varieties Chinese jujubes with contents > 1% (Table 1) were selected for analysis. As shown in Figure 3, the total contribution of PC1 (68.3%) and PC2 (20.2%) was 88.5%, and the result was in agreement with that of Figure 2. This indicates that the polyphenols with contents > 1% (Table 1) could accurately represent the whole profiles of the identified polyphenols (Table S1) in the 15 varieties of Chinese jujubes. Figure 3 show that HZ was characterized by pectolinarin, erianin, wogonoside, schizandrol A, syringetin and camelliaside A, which had the highest contents (*p* < 0.05), respectively (Table 1), while XSHZ, NYDZ and RQHZ, with similar polyphenol profiles, were characterized by (+)-catechin, combretastatin A4 and tectorigenin. JSBZ, HMDZ, TZ, JCJZ and HPZ were close to each other in terms of their similar polyphenol profiles, which were characterized by galangin, isoferulic acid and hydroxysafflor yellow A (Figure 3, Table 1). Clearly, the polyphenol profiles of the 15 varieties of Chinese jujubes were significant, and the characteristic individual polyphenol profiles of each jujube sample could be accurately analyzed by PCA.

## 4. Conclusions

A total of 79 characteristic polyphenols were identified in 15 varieties of Chinese jujube, and 55 characteristic polyphenols, such as syringetin, spinosin, kaempferol and apigenin, were reported for the first time. Scopoletin (63.94% in LZYZ), pectolinarin (22.63% in HZ), taxifolin (19.69% in HZ), camelliaside A (18.82% in NYDZ), combretastatin A4 (16.25% in XSHZ) and *p*-coumaric acid (10.67% in JSXZ) contributed greatly to the total polyphenol contents of the jujubes and presented significant difference in the 15 varieties of Chinese jujubes. Among these jujubes, HZ was characterized by pectolinarin, erianin, wogonoside, schizandrol A, syringetin and camelliaside, while XSHZ, NYDZ and RQHZ, with similar polyphenol profiles, were characterized by (+)-catechin, combretastatin A4 and tectorigenin. JSBZ, HMDZ, TZ, JCJZ and HPZ had similar polyphenol profiles of galangin, isoferulic acid and hydroxysafflor yellow A. Moreover, the differences in the polyphenol profiles and characteristic individual polyphenols of the 15 varieties of Chinese jujubes were accurately analyzed by PCA. The results of this study can provide reference for further research on the nutrition and function of Chinese jujubes.

## Figures and Tables

**Table 1:** Content of individual polyphenol (>1%) in 15 varieties of Chinese jujubes.

Compounds	Relative Content (%)														
	JSXZ	NHDZ	SZ	HMDZ	HZ	LZYZ	JSBZ	ZHDZ	TZ	XSHZ	RQHZ	JCJZ	HTDZ	HPZ	NYDZ
Phloretin	1.08 ± 0.02 h	0.50 ± 0.01 c	0.56 ± 0.01 d	0.85 ± 0.03 fg	0.27 ± 0.01 b	0.31 ± 0.00 c	0.10 ± 0.00 a	0.79 ± 0.01 f	0.70 ± 0.02 e	0.89 ± 0.01 g	0.25 ± 0.01 b	1.10 ± 0.03 h	0.61 ± 0.02 d	1.05 ± 0.01 h	0.52 ± 0.00 c
Ellagic acid	1.28 ± 0.04 j	0.12 ± 0.00 b	0.21 ± 0.01 cd	0.29 ± 0.01 f	0.10 ± 0.00 a	0.23 ± 0.00 de	0.10 ± 0.00 a	0.18 ± 0.01 c	0.13 ± 0.01 b	0.25 ± 0.01 e	0.30 ± 0.00 f	0.65 ± 0.02 h	1.24 ± 0.04 j	0.83 ± 0.04 i	0.35 ± 0.01 g
*p*-Coumaric acid	10.67 ± 0.35 j	3.68 ± 0.11 d	4.14 ± 0.15 e	5.29 ± 0.09 f	0.31 ± 0.02 a	3.83 ± 0.12 de	5.35 ± 0.20 f	6.95 ± 0.19 h	10.40 ± 0.33 j	3.26 ± 0.14 c	7.32 ± 0.17 h	6.06 ± 0.08 g	8.12 ± 0.26 i	10.30 ± 0.41 j	2.77 ± 0.05 b
Pectolinarin *	1.62 ± 0.02 c	0.46 ± 0.01 a	0.48 ± 0.00 a	1.63 ± 0.03 c	22.63 ± 0.72 i	0.85 ± 0.03 b	2.88 ± 0.10 d	1.56 ± 0.02 c	0.89 ± 0.03 b	9.27 ± 0.38 h	4.71 ± 0.21 f	3.79 ± 0.05 e	7.22 ± 0.09 g	3.80 ± 0.04 e	4.03 ± 0.12 e
Baohuoside II *	3.05 ± 0.07 h	2.11 ± 0.03 d	0.62 ± 0.01 a	2.37 ± 0.02 ef	3.00 ± 0.04 h	0.98 ± 0.01 b	2.31 ± 0.00 e	2.97 ± 0.05 h	1.26 ± 0.04 c	4.59 ± 0.15 i	5.81 ± 0.19 j	2.50 ± 0.03 fg	2.69 ± 0.05 g	2.16 ± 0.01 d	2.57 ± 0.04 g
Syringetin *	0.31 ± 0.00 j	0.02 ± 0.00 a	0.08 ± 0.01 f	0.11 ± 0.00 h	7.04 ± 0.18 k	0.02 ± 0.00 a	0.03 ± 0.00 b	0.08 ± 0.00 f	0.04 ± 0.00 c	0.09 ± 0.00 g	0.10 ± 0.00 h	0.07 ± 0.00 e	0.12 ± 0.00 i	0.06 ± 0.00 d	0.07 ± 0.00 e
Coumarin	0.45 ± 0.01 f	0.20 ± 0.00 d	0.16 ± 0.01 bc	0.15 ± 0.00 b	0.08 ± 0.00 a	0.49 ± 0.02 g	0.29 ± 0.00 e	0.40 ± 0.01 e	0.15 ± 0.01 a	0.17 ± 0.00 c	0.17 ± 0.01 c	0.85 ± 0.02 h	3.00 ± 0.12 j	1.35 ± 0.03 i	0.44 ± 0.01 ef
Morin Hydrate *	1.64 ± 0.04 h	0.35 ± 0.01 a	0.32 ± 0.01 a	1.39 ± 0.03 g	1.38 ± 0.02 g	0.41 ± 0.01 b	0.65 ± 0.02 d	1.01 ± 0.01 e	0.51 ± 0.01 c	1.64 ± 0.05 h	1.43 ± 0.02 g	1.78 ± 0.03 h	1.34 ± 0.01 g	1.09 ± 0.02 ef	1.10 ± 0.00 f
Hydroxysafflor yellow A *	0.12 ± 0.00 i	0.02 ± 0.00 a	0.03 ± 0.00 b	1.10 ± 0.02 j	0.05 ± 0.00 d	0.04 ± 0.00 c	0.02 ± 0.00 a	0.10 ± 0.00 g	0.05 ± 0.00 d	0.08 ± 0.00 f	0.11 ± 0.00 h	0.07 ± 0.00 e	0.08 ± 0.00 f	0.07 ± 0.00 e	0.11 ± 0.00 h
Taxifolin	15.86 ± 0.85 f	4.75 ± 0.32 a	4.64 ± 0.21 a	11.78 ± 0.47 de	19.69 ± 0.73 g	6.67 ± 0.59 b	10.38 ± 0.48 cd	13.34 ± 0.62 e	9.60 ± 0.52 c	15.72 ± 0.83 f	17.96 ± 0.91 fg	10.92 ± 0.35 cd	13.32 ± 0.27 e	11.16 ± 0.44 d	0.15 ± 0.00 e
Combretastatin A4 *	4.10 ± 0.12 cd	2.39 ± 0.09 b	0.90 ± 0.03 a	3.76 ± 0.17 c	6.10 ± 0.25 g	2.42 ± 0.16 b	5.62 ± 0.28 fg	7.81 ± 0.19 h	2.40 ± 0.05 b	16.25 ± 0.63 i	4.73 ± 0.11 e	4.31 ± 0.06 d	5.30 ± 0.03 f	5.74 ± 0.14 fg	0.06 ± 0.00 c
5-O-Caffeoylshikimic acid *	3.42 ± 0.12 m	0.08 ± 0.00 a	1.42 ± 0.03 k	0.26 ± 0.00 g	0.17 ± 0.01 c	0.98 ± 0.01 j	0.13 ± 0.00 b	0.88 ± 0.02 i	0.13 ± 0.00 b	0.33 ± 0.01 h	3.03 ± 0.04 l	0.21 ± 0.00 e	0.18 ± 0.01 cd	0.20 ± 0.00 de	0.29 ± 0.00 h
Rosmarinic acid	0.78 ± 0.01 f	0.15 ± 0.00 b	0.60 ± 0.01 e	0.60 ± 0.01 e	0.13 ± 0.00 a	0.36 ± 0.01 d	0.29 ± 0.01 c	5.09 ± 0.12 m	1.35 ± 0.03 j	2.04 ± 0.02 l	1.66 ± 0.02 k	0.98 ± 0.03 h	1.13 ± 0.01 i	0.90 ± 0.00 g	0.05 ± 0.00 d
Nobiletin	1.09 ± 0.02 jk	0.43 ± 0.01 ef	0.16 ± 0.00 b	1.11 ± 0.05 k	0.27 ± 0.01 d	0.25 ± 0.01 d	0.05 ± 0.01 a	0.22 ± 0.01 c	1.04 ± 0.03 j	0.45 ± 0.01 f	0.58 ± 0.01 h	0.68 ± 0.02 i	0.40 ± 0.01 e	0.45 ± 0.01 f	0.03 ± 0.00 c
Isomucronulatol *	1.02 ± 0.02 h	0.22 ± 0.00 b	0.22 ± 0.00 b	0.66 ± 0.01 f	0.20 ± 0.00 a	0.25 ± 0.01 c	0.20 ± 0.00 a	0.83 ± 0.02 g	0.52 ± 0.02 d	1.14 ± 0.03 i	0.64 ± 0.01 f	0.58 ± 0.01 e	0.49 ± 0.01 d	0.83 ± 0.02 g	0.03 ± 0.00 c
Erianin *	0.20 ± 0.00 g	0.12 ± 0.00 g	0.03 ± 0.00 a	0.07 ± 0.00 d	1.08 ± 0.02 h	0.03 ± 0.00 a	0.10 ± 0.00 f	0.07 ± 0.00 d	0.06 ± 0.00 c	0.15 ± 0.00 e	0.09 ± 0.00 e	0.22 ± 0.01 g	0.12 ± 0.00 g	0.17 ± 0.01 f	0.06 ± 0.00 e
Wogonoside *	0.04 ± 0.00 a	0.18 ± 0.00 h	0.05 ± 0.00 b	0.14 ± 0.00 g	7.26 ± 0.28 n	0.08 ± 0.00 c	0.12 ± 0.00 f	0.15 ± 0.01 g	0.11 ± 0.00 e	1.42 ± 0.05 m	0.09 ± 0.00 d	0.28 ± 0.01 k	0.34 ± 0.00 l	0.20 ± 0.00 i	0.03 ± 0.00 c
Scopoletin	26.26 ± 0.73 d	72.60 ± 1.04 k	76.96 ± 0.98 k	47.48 ± 1.16 h	5.88 ± 0.17 a	63.94 ± 0.55 j	53.50 ± 0.70 i	34.90 ± 1.01 f	52.02 ± 1.37 hi	15.68 ± 0.52 b	20.44 ± 0.77 c	43.46 ± 1.12 gh	30.00 ± 0.27 e	42.73 ± 0.85 g	0.03 ± 0.00 c
Camelliaside A *	0.08 ± 0.00 e	0.08 ± 0.00 e	0.02 ± 0.00 a	0.03 ± 0.00 b	3.28 ± 0.12 h	0.02 ± 0.00 a	0.04 ± 0.00 c	0.03 ± 0.00 b	0.05 ± 0.00 d	0.15 ± 0.00 g	0.11 ± 0.00 f	0.08 ± 0.00 e	0.05 ± 0.00 d	0.05 ± 0.00 d	18.82 ± 0.62 g
Isoliquiritin *	0.72 ± 0.01 g	0.74 ± 0.02 g	0.25 ± 0.01 c	0.23 ± 0.01 c	0.01 ± 0.00 a	0.73 ± 0.01 g	0.50 ± 0.02 e	0.82 ± 0.02 h	0.32 ± 0.01 d	0.11 ± 0.01 b	0.96 ± 0.02 i	1.79 ± 0.04 j	5.13 ± 0.34 l	2.29 ± 0.18 k	4.55 ± 0.09 de
Galangin *	0.74 ± 0.02 h	0.26 ± 0.01 d	0.14 ± 0.00 a	0.14 ± 0.00 a	0.26 ± 0.00 d	2.71 ± 0.05 k	0.14 ± 0.00 a	0.37 ± 0.01 f	1.22 ± 0.03 i	0.18 ± 0.00 c	0.33 ± 0.00 e	0.56 ± 0.02 g	2.31 ± 0.05 j	0.41 ± 0.01 f	0.24 ± 0.00 f
Ferulic acid	4.31 ± 0.07 j	0.73 ± 0.02 c	0.59 ± 0.01 b	2.95 ± 0.08 i	0.18 ± 0.00 a	1.31 ± 0.04 e	1.60 ± 0.03 f	1.41 ± 0.02 e	0.92 ± 0.02 d	2.89 ± 0.07 i	2.60 ± 0.05 h	2.38 ± 0.04 h	1.61 ± 0.05	1.87 ± 0.02 g	1.41 ± 0.02 j
Isoferulic acid *	0.74 ± 0.01 j	0.27 ± 0.01 e	0.14 ± 0.00 a	0.15 ± 0.01 ab	0.27 ± 0.01 e	2.71 ± 0.06 m	0.21 ± 0.01 d	0.37 ± 0.00 g	1.22 ± 0.03 k	0.18 ± 0.00 c	0.34 ± 0.01 f	0.56 ± 0.01 i	2.31 ± 0.05 l	0.40 ± 0.01 h	0.49 ± 0.00 g
Demethylnobiletin *	0.47 ± 0.01 d	0.96 ± 0.02 h	0.89 ± 0.01 g	0.96 ± 0.01 h	0.02 ± 0.00 a	1.11 ± 0.03 i	0.29 ± 0.00 c	1.21 ± 0.01 j	0.65 ± 0.02 e	1.10 ± 0.02 i	0.13 ± 0.00 b	0.81 ± 0.01 f	0.43 ± 0.01 d	0.80 ± 0.02 f	0.66 ± 0.02 j
Kaempferol *	0.90 ± 0.01 h	0.34 ± 0.01 c	0.18 ± 0.00 a	0.44 ± 0.02 d	2.10 ± 0.05 k	0.33 ± 0.01 c	0.70 ± 0.01 f	0.84 ± 0.02 h	0.44 ± 0.01 d	1.03 ± 0.02 i	1.21 ± 0.02 j	0.52 ± 0.01 e	0.54 ± 0.02 e	0.29 ± 0.01 b	0.56 ± 0.01 h
(+)-Catechin	0.06 ± 0.00 d	0.05 ± 0.00 c	0.02 ± 0.00 a	0.05 ± 0.00 c	0.05 ± 0.00 c	0.02 ± 0.00 a	0.02 ± 0.00 a	0.36 ± 0.01 f	0.05 ± 0.00 c	1.08 ± 0.02 g	0.07 ± 0.00 d	0.37 ± 0.00 f	0.04 ± 0.00 b	0.05 ± 0.00 c	0.78 ± 0.01 g
Tectorigenin *	3.37 ± 0.05 g	1.74 ± 0.02 cd	0.75 ± 0.02 a	1.97 ± 0.05 e	2.68 ± 0.07 f	1.34 ± 0.01 b	5.40 ± 0.04 j	2.72 ± 0.10 f	1.38 ± 0.06 b	5.04 ± 0.06 i	4.60 ± 0.03 h	1.87 ± 0.02 de	1.82 ± 0.02 d	1.61 ± 0.03 c	0.05 ± 0.00 b
Rhapontigenin *	1.07 ± 0.03 k	0.52 ± 0.01 h	0.17 ± 0.00 b	0.58 ± 0.01 i	0.01 ± 0.00 a	0.24 ± 0.00 c	0.35 ± 0.01 e	0.31 ± 0.00 d	0.39 ± 0.01 f	0.46 ± 0.01 g	0.82 ± 0.02 j	1.47 ± 0.03 l	0.31 ± 0.00 d	0.50 ± 0.01 gh	0.14 ± 0.00 f
Epicatechin	2.05 ± 0.06 j	0.31 ± 0.00 b	0.12 ± 0.00 a	2.21 ± 0.04 j	0.43 ± 0.01 d	0.50 ± 0.01 e	3.34 ± 0.07 k	0.55 ± 0.01 f	1.13 ± 0.03 h	1.21 ± 0.02 h	5.49 ± 0.11 l	0.83 ± 0.02 g	0.40 ± 0.01 d	0.35 ± 0.00 c	0.62 ± 0.01 f
Schisanhenol *	1.28 ± 0.03 j	0.70 ± 0.00 f	0.77 ± 0.01 gh	0.99 ± 0.02 i	0.04 ± 0.00 a	0.81 ± 0.02 h	0.29 ± 0.00 c	0.62 ± 0.01 e	0.23 ± 0.00 b	0.50 ± 0.02 d	0.74 ± 0.01 fg	0.60 ± 0.01 e	0.63 ± 0.02 e	0.52 ± 0.01 d	0.58 ± 0.02 j
Schizandrol A *	0.04 ± 0.00 b	0.05 ± 0.00 c	0.03 ± 0.00 a	0.04 ± 0.00 b	6.96 ± 0.11 j	0.05 ± 0.00 c	0.08 ± 0.00 e	0.08 ± 0.00 e	0.07 ± 0.00 d	0.15 ± 0.00 i	0.04 ± 0.00 b	0.10 ± 0.00 g	0.09 ± 0.00 f	0.07 ± 0.00 d	0.67 ± 0.02 l
Procyanidin B2	1.76 ± 0.02 d	0.27 ± 0.00 b	1.15 ± 0.02 c	2.63 ± 0.05 g	0.15 ± 0.00 a	2.29 ± 0.04 f	0.27 ± 0.00 b	7.06 ± 0.16 j	5.62 ± 0.08 i	1.98 ± 0.04 e	4.64 ± 0.11 h	1.91 ± 0.06 e	2.39 ± 0.07 fg	2.00 ± 0.03 e	0.21 ± 0.01 fg
Cyanidin 3-rutinoside *	1.70 ± 0.03 j	0.07 ± 0.00 a	0.22 ± 0.00 g	0.16 ± 0.00 f	0.13 ± 0.00 d	0.24 ± 0.00 h	0.12 ± 0.00 c	0.13 ± 0.00 d	0.08 ± 0.00 b	0.14 ± 0.00 e	0.24 ± 0.00 h	0.26 ± 0.00 i	0.27 ± 0.01 i	0.27 ± 0.00 i	0.10 ± 0.00 f

JSXZ, Jin Si Xiao Zao; NHDZ, Nei Huang Da Zao; SZ, Suan Zao; HMDZ, Ha Mi Da Zao; HZ, Hui Zao; LZYZ, Lin Ze Yu Zao; JSBZ, Ji Shan Ban Zao; ZHDZ, Zan Huang Da Zao; TZ, Tan Zao; XSHZ, Xi Sha Hong Zao; RQHZ, Ruo Qiang Hui Zao; JCJZ, Jiao Cheng Jun Zao; HTDZ, He Tian Da Zao; HPZ, Hu Ping Zao; NYDZ, Ning Yang Da Zao. Mean values with different lower-case letters in the same line correspond to significant differences at *p* < 0.05. Data are represented as the mean ± SD (standard deviation). Compounds marked with “*” were reported in jujubes for the first time.

## Data Availability

The original contributions presented in the study are included in the article/Supplementary Materials, further inquiries can be directed to the corresponding author.

## Supplementary Materials

The following supporting information can be downloaded at: https://www.mdpi.com/article/10.3390/metabo14120661/s1, Table S1: Identification of individual polyphenol compound in 15 varieties of Chinese jujubes; Table S2: Content of individual polyphenol in 15 varieties of Chinese jujubes.
