# Supplementary material for: Characteristic Polyphenols in 15 Varieties of Chinese Jujubes Based on Metabolomics
Source: Metabolites. 2024 Nov 28;14(12):661. doi: 10.3390/metabo14120661 (PMC11676182; doi:10.3390/metabo14120661)
Supplement: Supplementary file 1 [file metabolites-14-00661-s001.zip › metabolites-3309918-supplementary.pdf]

Table S1 Identification of individual polyphenol compound in 15 varieties of Chinese jujubes.

NO.	RT	Compounds	*m/z*	CAS[#]	Molecular weight	Molecular formula	Molecular structure
1	0.903	Phloretin	273.07629	60-82-2	274.08357	$C_{15}H_{14}O_5$	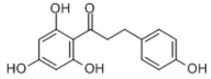
2	1.226	Ellagic acid	303.01433	476-66-4	302.00721	$C_{14}H_6O_8$	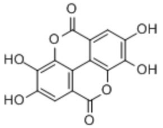
3	1.682	*p*-Coumaric acid	165.05476	501-98-4	164.04749	$C_9H_8O_3$	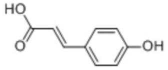
4	1.823	Pectolinarin *	621.17999	28978-02-1	622.18727	$C_{29}H_{34}O_{15}$	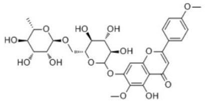
5	1.949	Baohuoside II *	501.17523	55395-07-8	500.16795	$C_{26}H_{28}O_{10}$	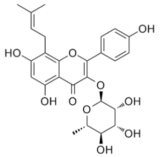
6	2.084	Syringetin *	347.07495	4423-37-4	346.06767	$C_{17}H_{14}O_8$	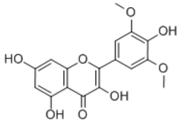
7	2.400	Coumarin	147.04413	91-64-5	146.03685	$C_9H_6O_2$	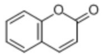
8	4.797	Eupatilin *	345.09571	22368-21-4	344.08844	$C_{18}H_{16}O_7$	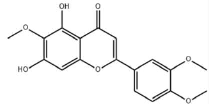

9	4.873	Morin Hydrate *	319.04561	654055-01-3	320.05289	$C_{15}H_{12}O_{8}$	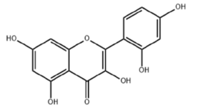
10	5.001	Procyanidin B1	577.13606	20315-25-7	578.14333	$C_{30}H_{26}O_{12}$	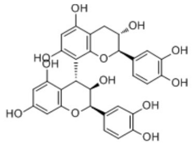
11	5.069	Hydroxysafflor yellow A *	611.16234	78281-02-4	612.16962	$C_{27}H_{32}O_{16}$	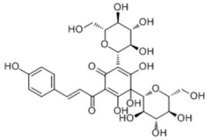
12	5.091	Catechin	291.08643	7295-85-4	290.07913	$C_{15}H_{14}O_{6}$	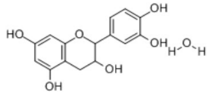
13	5.123	Typhaneoside *	769.22165	104472-68-6	770.22893	$C_{34}H_{42}O_{20}$	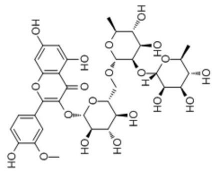
14	5.140	Resveratrol	229.08609	501-36-0	228.0797	$C_{14}H_{12}O_{3}$	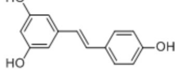
15	5.173	Myricetin 3-O-galactoside *	479.08331	15648-86-9	480.09058	$C_{21}H_{20}O_{13}$	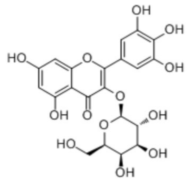

16	5.177	Gallocatechin gallate *	457.07965	4233-96-9	458.08693	$C_{22} H_{18} O_{11}$	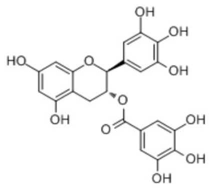
17	5.183	Trilobatin	459.12863	4192-90-9	436.13899	$C_{21} H_{24} O_{10}$	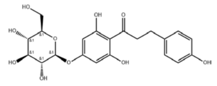
18	5.193	Esculin *	339.07202	531-75-9	340.07934	$C_{15} H_{16} O_{9}$	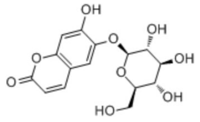
19	5.263	Astilbin *	449.10882	29838-67-3	450.1161	$C_{21} H_{22} O_{11}$	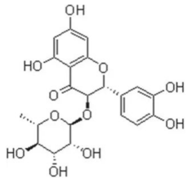
20	5.279	Spinosin *	607.16498	72063-39-9	608.17225	$C_{28} H_{32} O_{15}$	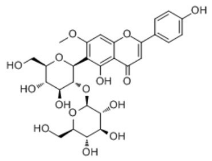
21	5.284	Taxifolin	322.09229	480-18-2	304.0584	$C_{15} H_{12} O_{7}$	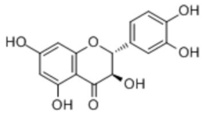
22	5.286	Combretastatin A4 *	317.13859	117048-59-6	316.13131	$C_{18} H_{20} O_{5}$	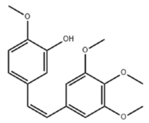

23	5.294	5-O-Caffeoylshikimic acid *	335.07719	73263-62-4	336.0843	$C_{16} H_{16} O_{8}$	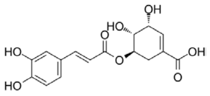
24	5.367	Rosmarinic acid	361.09134	20283-92-5	360.08406	$C_{18} H_{16} O_{8}$	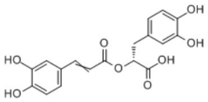
25	5.374	Nobiletin	403.13704	478-01-3	402.12977	$C_{21} H_{22} O_{8}$	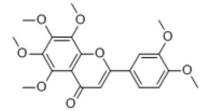
26	5.385	Camelliaside B *	725.19428	131573-90-5	726.20154	$C_{32} H_{38} O_{19}$	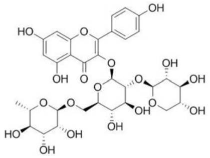
27	5.419	Isosakuranetin *	309.07254	480-43-3	286.0833	$C_{16} H_{14} O_{5}$	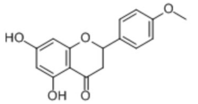
28	5.423	Isomucronulatol *	301.10794	64474-51-7	302.11522	$C_{17} H_{18} O_{5}$	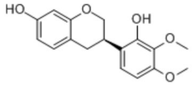
29	5.428	Erianin *	654.32776	95041-90-0	318.14702	$C_{18} H_{22} O_{5}$	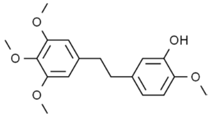
30	5.428	Puerarin *	417.11602	3681-99-0	416.10874	$C_{21} H_{20} O_{9}$	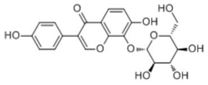
31	5.432	Baohuoside I *	513.17767	113558-15-9	514.18409	$C_{27} H_{30} O_{10}$	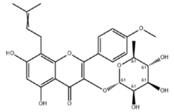

32	5.433	Naringenin chalcone *	290.10245	736-92-5	272.06858	$C_{15}H_{12}O_5$	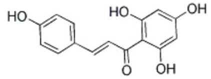
33	5.433	Narirutin *	579.1719	14259-46-2	580.17901	$C_{27}H_{32}O_{14}$	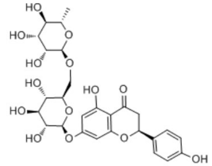
34	5.439	Procyanidin A2 *	577.13489	41743-41-3	576.12762	$C_{30}H_{24}O_{12}$	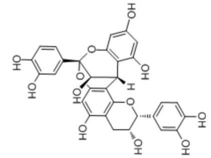
35	5.443	Kuwanon A *	419.14856	62949-77-3	420.15584	$C_{25}H_{24}O_6$	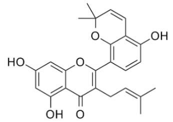
36	5.449	Rutin	609.14555	153-18-4	610.15283	$C_{27}H_{30}O_{16}$	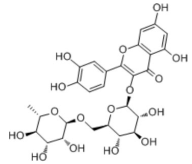
37	5.451	Wogonoside *	459.09424	51059-44-0	460.10151	$C_{22}H_{20}O_{11}$	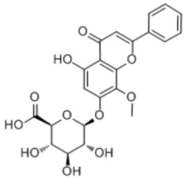
38	5.455	6-Methylcoumarin *	161.05983	92-48-8	160.05255	$C_{10}H_8O_2$	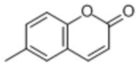

39	5.480	Quercetin	303.04997	117-39-5	302.04269	$C_{15}H_{10}O_7$	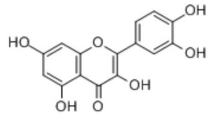
40	5.481	Scopoletin	193.04981	92-61-5	192.04253	$C_{10}H_8O_4$	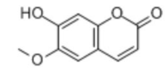
41	5.484	Camelliaside A *	755.20474	135095-52-2	756.21209	$C_{33}H_{40}O_{20}$	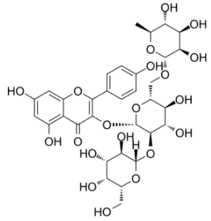
42	5.503	Isoliquiritin *	419.13249	5041-81-6	418.12586	$C_{21}H_{22}O_9$	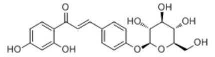
43	5.518	Galangin *	288.08679	548-83-4	270.05292	$C_{15}H_{10}O_5$	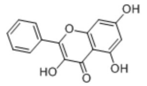
44	5.520	Ferulic acid	195.06558	1135-24-6	194.05812	$C_{10}H_{10}O_4$	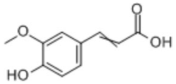
45	5.525	Isoferulic acid *	193.05041	537-73-5	194.05769	$C_{10}H_{10}O_4$	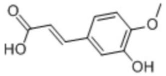
46	5.529	Demethylnobiletin *	389.12188	2174-59-6	388.1146	$C_{20}H_{20}O_8$	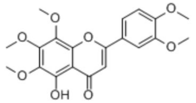
47	5.536	Eriodictyol	287.05578	552-58-9	288.0633	$C_{15}H_{12}O_6$	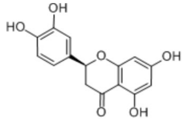

48	5.539	Kaempferol *	287.05482	520-18-3	286.04754	$C_{15}H_{10}O_6$	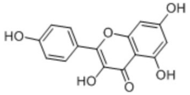
49	5.542	Myricetin	319.04515	529-44-2	318.03786	$C_{15}H_{10}O_8$	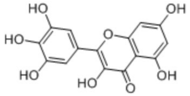
50	5.544	(+)-Catechin	291.08569	154-23-4	290.07851	$C_{15}H_{14}O_6$	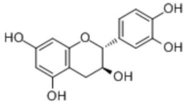
51	5.559	Polydatin *	435.12943	27208-80-6	390.1311	$C_{20}H_{22}O_8$	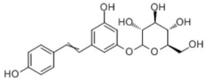
52	5.564	Phloridzin	435.12955	60-81-1	436.13685	$C_{21}H_{24}O_{10}$	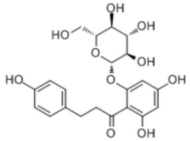
53	5.660	Esculetin *	179.0341	305-01-1	178.02683	$C_9H_6O_4$	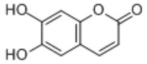
54	5.667	Angelol B *	377.15796	83156-04-1	376.15069	$C_{20}H_{24}O_7$	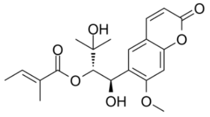
55	5.700	Casticin *	375.10614	479-91-4	374.09886	$C_{19}H_{18}O_8$	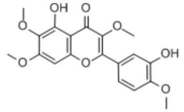
56	5.713	Trifolirhizin *	445.11383	6807-83-6	446.1211	$C_{22}H_{22}O_{10}$	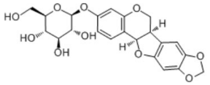

57	5.771	Pelargonidin chloride *	305.02195	134-04-3	306.02923	$C_{15} H_{11} Cl O_{5}$	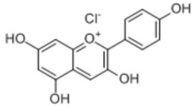
58	5.816	Naringin Dihydrochalcone *	581.1886	18916-17-1	582.19588	$C_{27} H_{34} O_{14}$	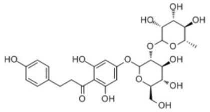
59	5.851	Isorhamnetin	315.05067	480-19-3	316.05788	$C_{16} H_{12} O_{7}$	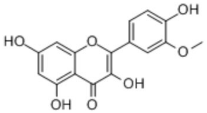
60	5.870	Luteolin	285.04012	491-70-3	286.04734	$C_{15} H_{10} O_{6}$	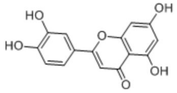
61	5.894	Hesperetin 5-O-glucoside *	463.12454	69651-80-5	464.13182	$C_{22} H_{24} O_{11}$	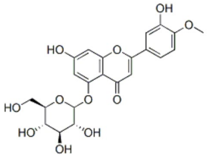
62	5.901	Naringenin	271.06073	480-41-1	272.06801	$C_{15} H_{12} O_{5}$	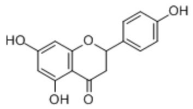
63	5.906	Rhapontin *	419.13412	155-58-8	420.14143	$C_{21} H_{24} O_{9}$	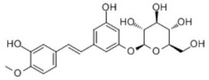
64	5.989	Orientin *	447.0936	28608-75-5	448.10088	$C_{21} H_{20} O_{11}$	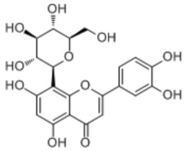

65	5.995	Delphinidin chloride *	337.01062	16727-30-3	338.0179	$C_{15}H_{11}ClO_7$	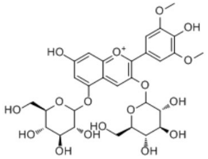
66	6.055	Schisantherin A *	535.19751	58546-56-8	536.20485	$C_{30}H_{32}O_9$	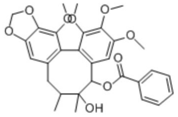
67	6.062	Tectorigenin *	299.05575	548-77-6	300.063	$C_{16}H_{12}O_6$	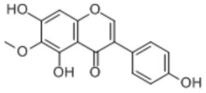
68	6.104	Apigenin *	269.04508	520-36-5	270.0523	$C_{15}H_{10}O_5$	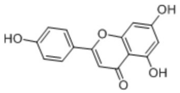
69	6.167	Schisandrin A *	417.22619	61281-38-7	416.21891	C_{24} H32 O6	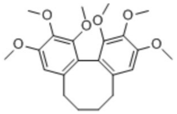
70	6.457	Isoliquiritigenin *	255.06538	961-29-5	256.07266	$C_{15}H_{12}O_4$	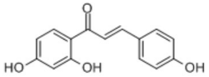
71	6.499	Rhapontigenin *	257.08129	500-65-2	258.08857	$C_{15}H_{14}O_4$	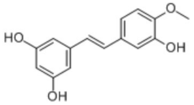
72	6.563	Epicatechin	289.07126	490-46-0	290.07837	$C_{15}H_{14}O_6$	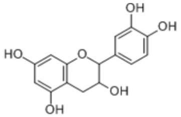

73	6.625	Chrysin *	253.04999	480-40-0	254.05727	$C_{15} H_{10} O_4$	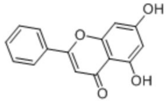
74	6.878	Schisanhenol *	403.20968	69363-14-0	402.2024	$C_{23} H_{30} O_6$	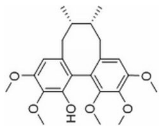
75	7.412	Schizandrol A *	433.22003	7432-28-2	432.21276	$C_{24} H_{32} O_7$	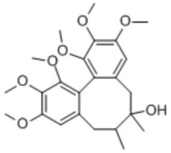
76	9.341	Procyanidin B2	577.13464	29106-49-8	578.14192	$C_{30} H_{26} O_{12}$	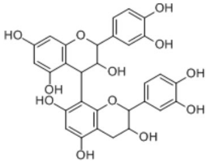
77	9.574	Schisandrin C *	385.16544	63101-33-5	384.15766	$C_{22} H_{24} O_6$	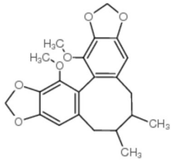
78	9.838	Cyanidin 3-rutinoside *	594.1579	28338-59-2	595.16517	$C_{27} H_{31} O_{15}$	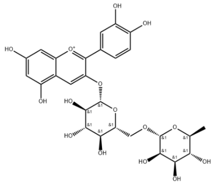
79	11.651	Coumarin 151 *	230.04239	53518-15-3	229.03511	$C_{10} H_6 F_3 N O_2$	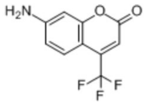

Table S2 Content of individual polyphenol in 15 varieties of Chinese jujubes.

Compounds	Relative content (%)														
	JSXZ	NHDZ	SZ	HMDZ	HZ	LZYZ	JSBZ	ZHDZ	TZ	XSHZ	RQHZ	JCJZ	HTDZ	HPZ	NYDZ
Phloretin	1.08±0.02h	0.50±0.01c	0.56±0.01d	0.85±0.03fg	0.27±0.01b	0.31±0.00c	0.10±0.00a	0.79±0.01f	0.70±0.02e	0.89±0.01g	0.25±0.01b	1.10±0.03h	0.61±0.02d	1.05±0.01h	0.52±0.00c
Ellagic acid	1.28±0.04j	0.12±0.00b	0.21±0.01cd	0.29±0.01f	0.10±0.00a	0.23±0.00de	0.10±0.00a	0.18±0.01c	0.13±0.01b	0.25±0.01e	0.30±0.00f	0.65±0.02h	1.24±0.04j	0.83±0.04i	0.35±0.01g
p-Coumaric acid	10.67±0.35j	3.68±0.11d	4.14±0.15e	5.29±0.09f	0.31±0.02a	3.83±0.12de	5.35±0.20f	6.95±0.19h	10.40±0.33j	3.26±0.14c	7.32±0.17h	6.06±0.08g	8.12±0.26i	10.30±0.41j	2.77±0.05b
Pectolinarin	1.62±0.02c	0.46±0.01a	0.48±0.00a	1.63±0.03c	22.63±0.72i	0.85±0.03b	2.88±0.10d	1.56±0.02c	0.89±0.03b	9.27±0.38h	4.71±0.21f	3.79±0.05e	7.22±0.09g	3.80±0.04e	4.03±0.12e
Baohuoside II	3.05±0.07h	2.11±0.03d	0.62±0.01a	2.37±0.02ef	3.00±0.04h	0.98±0.01b	2.31±0.00e	2.97±0.05h	1.26±0.04c	4.59±0.15i	5.81±0.19j	2.50±0.03fg	2.69±0.05g	2.16±0.01d	2.57±0.04g
Syringetin	0.31±0.00j	0.02±0.00a	0.08±0.01f	0.11±0.00h	7.04±0.18k	0.02±0.00a	0.03±0.00b	0.08±0.00f	0.04±0.00c	0.09±0.00g	0.10±0.00h	0.07±0.00e	0.12±0.00i	0.06±0.00d	0.07±0.00e
Coumarin	0.45±0.01f	0.20±0.00d	0.16±0.01bc	0.15±0.00b	0.08±0.00a	0.49±0.02g	0.29±0.00e	0.40±0.01e	0.15±0.01a	0.17±0.00c	0.17±0.01c	0.85±0.02h	3.00±0.12j	1.35±0.03i	0.44±0.01ef
Eupatilin	0.43±0.01j	0.19±0.00h	0.51±0.01k	0.04±0.00d	0.02±0.00b	0.50±0.01k	0.01±0.00a	0.19±0.00h	0.15±0.00f	0.44±0.01j	0.17±0.01g	0.03±0.00c	0.33±0.02i	0.07±0.00e	0.02±0.00b
Morin Hydrate	1.64±0.04h	0.35±0.01a	0.32±0.01a	1.39±0.03g	1.38±0.02g	0.41±0.01b	0.65±0.02d	1.01±0.01e	0.51±0.01c	1.64±0.05h	1.43±0.02g	1.78±0.03h	1.34±0.01g	1.09±0.02ef	1.10±0.00f
Procyanidin B1	0.48±0.01i	0.11±0.00d	0.20±0.00c	0.08±0.00c	0.34±0.01g	0.27±0.01e	0.04±0.00a	0.37±0.01gh	0.82±0.02j	0.31±0.00f	0.40±0.01h	0.07±0.00b	0.39±0.02h	0.22±0.00d	1.00±0.02k
Hydroxysafflor yellow A	0.12±0.00i	0.02±0.00a	0.03±0.00b	1.10±0.02j	0.05±0.00d	0.04±0.00c	0.02±0.00a	0.10±0.00g	0.05±0.00d	0.08±0.00f	0.11±0.00h	0.07±0.00e	0.08±0.00f	0.07±0.00e	0.11±0.00h

Catechin	0.18± 0.00f	0.07± 0.00d	0.16± 0.01e	0.35± 0.01h	0.03± 0.00b	0.02± 0.00a	0.07± 0.00d	0.18± 0.00f	0.02± 0.00a	0.35± 0.01h	0.29± 0.01g	0.05± 0.00c	0.27± 0.01g	0.03± 0.00b	0.15± 0.00e
Typhaneoside	0.06± 0.00c	0.02± 0.00a	0.02± 0.00a	0.09± 0.01f	0.71± 0.01h	0.02± 0.00a	0.05± 0.00c	0.08± 0.00e	0.03± 0.00b	0.10± 0.00g	0.11± 0.01g	0.07± 0.00d	0.05± 0.00c	0.04± 0.00b	0.06± 0.00c
Resveratrol	0.06± 0.00c	0.02± 0.00a	0.05± 0.00b	0.15± 0.00f	0.02± 0.00a	0.02± 0.00a	0.02± 0.00a	0.06± 0.00c	0.16± 0.01f	0.06± 0.00c	0.09± 0.00d	0.11± 0.00e	0.09± 0.00d	0.25± 0.01g	0.29± 0.00h
Myricetin 3-O-galactoside	0.05± 0.00d	0.01± 0.00a	0.01± 0.00a	0.06± 0.00e	0.04± 0.00c	0.01± 0.00a	0.03± 0.00b	0.09± 0.00g	0.04± 0.00c	0.07± 0.00f	0.43± 0.02h	0.04± 0.00c	0.04± 0.00c	0.03± 0.00b	0.05± 0.00d
Gallocatechin gallate	0.10± 0.00g	0.03± 0.00c	0.01± 0.00a	0.04± 0.00d	0.24± 0.01h	0.01± 0.01a	0.09± 0.01f	0.03± 0.01c	0.02± 0.01b	0.05± 0.00e	0.05± 0.00e	0.05± 0.00e	0.03± 0.00c	0.03± 0.00c	0.03± 0.00c
Trilobatin	0.16± 0.00g	0.01± 0.00a	0.05± 0.00e	0.04± 0.00d	0.02± 0.00b	0.08± 0.00f	0.02± 0.00b	0.03± 0.00c	0.01± 0.00a	0.04± 0.00d	0.05± 0.00e	0.02± 0.00b	0.02± 0.00b	0.02± 0.00b	0.03± 0.00c
Esculin	0.09± 0.00g	0.03± 0.00c	0.01± 0.00a	0.10± 0.00h	0.02± 0.00b	0.03± 0.00c	0.05± 0.00d	0.06± 0.00e	0.03± 0.00c	0.11± 0.00i	0.17± 0.00j	0.03± 0.00c	0.05± 0.00d	0.07± 0.00f	0.06± 0.00e
Astilbin	0.03± 0.00c	0.01± 0.00a	0.01± 0.00a	0.04± 0.00d	0.03± 0.00c	0.01± 0.00a	0.02± 0.00b	0.03± 0.00c	0.02± 0.00b	0.09± 0.00e	0.04± 0.00d	0.02± 0.00b	0.02± 0.00b	0.02± 0.00b	0.03± 0.00c
Spinosin	0.03± 0.00c	0.01± 0.00a	0.02± 0.00b	0.03± 0.00c	0.03± 0.00c	0.02± 0.00b	0.02± 0.00b	0.04± 0.00d	0.02± 0.00b	0.10± 0.00f	0.07± 0.00e	0.03± 0.00c	0.02± 0.00b	0.02± 0.00b	0.03± 0.00c
Taxifolin	15.86± 0.85f	4.75± 0.32a	4.64± 0.21a	11.78± 0.47de	19.69± 0.73g	6.67± 0.59b	10.38± 0.48cd	13.34± 0.62e	9.60± 0.52c	15.72± 0.83f	17.96± 0.91fg	10.92± 0.35cd	13.32± 0.27e	11.16± 0.44d	18.82± 0.62g
Combretastatin A4	4.10± 0.12cd	2.39± 0.09b	0.90± 0.03a	3.76± 0.17c	6.10± 0.25g	2.42± 0.16b	5.62± 0.28fg	7.81± 0.19h	2.40± 0.05b	16.25± 0.63i	4.73± 0.11e	4.31± 0.06d	5.30± 0.03f	5.74± 0.14fg	4.55± 0.09de
5-O-Caffeoylshikimic acid	3.42± 0.12m	0.08± 0.00a	1.42± 0.03k	0.26± 0.00g	0.17± 0.01c	0.98± 0.01j	0.13± 0.00b	0.88± 0.02i	0.13± 0.00b	0.33± 0.01h	3.03± 0.04l	0.21± 0.00e	0.18± 0.01cd	0.20± 0.00de	0.24± 0.00f
Rosmarinic acid	0.78± 0.01f	0.15± 0.00b	0.60± 0.01e	0.60± 0.01e	0.13± 0.00a	0.36± 0.01d	0.29± 0.01c	5.09± 0.12m	1.35± 0.03j	2.04± 0.02l	1.66± 0.02k	0.98± 0.03h	1.13± 0.01i	0.90± 0.00g	1.41± 0.02j

Nobiletin	1.09±0.02jk	0.43±0.01ef	0.16±0.00b	1.11±0.05k	0.27±0.01d	0.25±0.01d	0.05±0.01a	0.22±0.01c	1.04±0.03j	0.45±0.01f	0.58±0.01h	0.68±0.02i	0.40±0.01e	0.45±0.01f	0.49±0.00g
Camelliaside B	0.47±0.01h	0.17±0.00b	0.16±0.00b	0.36±0.01g	0.28±0.00e	0.12±0.00a	0.63±0.01j	0.19±0.00c	0.24±0.00d	0.32±0.01f	0.37±0.01g	0.53±0.01i	0.65±0.01j	0.46±0.00h	0.66±0.02j
Isosakuranetin	0.68±0.02i	0.21±0.00c	0.20±0.00c	0.83±0.01j	0.12±0.00b	0.27±0.01d	0.11±0.00a	0.15±0.00c	0.30±0.01de	0.37±0.00f	0.31±0.00e	0.95±0.01k	0.38±0.01fg	0.41±0.01g	0.56±0.01h
Isomucronulatol	1.02±0.02h	0.22±0.00b	0.22±0.00b	0.66±0.01f	0.20±0.00a	0.25±0.01c	0.20±0.00a	0.83±0.02g	0.52±0.02d	1.14±0.03i	0.64±0.01f	0.58±0.01e	0.49±0.01d	0.83±0.02g	0.78±0.01g
Erianin	0.20±0.00g	0.12±0.00g	0.03±0.00a	0.07±0.00d	1.08±0.02h	0.03±0.00a	0.10±0.00f	0.07±0.00d	0.06±0.00c	0.15±0.00e	0.09±0.00e	0.22±0.01g	0.12±0.00g	0.17±0.01f	0.05±0.00b
Puerarin	0.07±0.00c	0.11±0.00e	0.09±0.00d	0.09±0.00d	0.02±0.00a	0.03±0.00b	0.29±0.01h	0.65±0.01j	0.31±0.00h	0.43±0.01i	0.16±0.00g	0.07±0.00c	0.09±0.00d	0.31±0.01h	0.14±0.00f
Baohuoside I	0.57±0.01ef	0.14±0.00a	0.16±0.00b	0.35±0.01d	0.95±0.01h	0.21±0.00c	0.22±0.01c	0.54±0.01e	0.38±0.01d	0.61±0.02f	0.67±0.02fg	0.68±0.01g	0.64±0.01fg	0.58±0.01ef	0.62±0.01f
Naringenin chalcone	0.50±0.01i	0.12±0.00c	0.15±0.00d	0.38±0.01fg	0.07±0.00a	0.12±0.00c	0.11±0.00b	0.40±0.01g	0.35±0.01f	0.67±0.01k	0.46±0.02h	0.42±0.01gh	0.18±0.00e	0.40±0.01g	0.58±0.02j
Narirutin	0.15±0.00g	0.04±0.00b	0.03±0.00a	0.82±0.01m	0.05±0.00c	0.09±0.00d	0.10±0.00e	0.17±0.00h	0.12±0.00f	0.24±0.01i	0.41±0.01k	0.29±0.00j	0.09±0.00d	0.23±0.00i	0.67±0.02l
Procyanidin A2	0.25±0.01h	0.07±0.00a	0.11±0.00b	0.22±0.00g	0.16±0.00d	0.18±0.00e	0.11±0.00b	0.28±0.01i	0.13±0.00c	0.30±0.01i	0.34±0.01j	0.19±0.00ef	0.17±0.01de	0.18±0.00e	0.21±0.01fg
Kuwanon A	0.16±0.00i	0.03±0.00a	0.04±0.00b	0.14±0.00g	0.05±0.00c	0.05±0.00c	0.04±0.00b	0.07±0.00d	0.09±0.00e	0.15±0.00h	0.16±0.00i	0.10±0.00f	0.20±0.01j	0.11±0.01f	0.10±0.00f
Rutin	0.13±0.00f	0.02±0.00a	0.02±0.00a	0.23±0.00h	0.07±0.00c	0.02±0.00a	0.07±0.00c	0.09±0.00d	0.02±0.00a	0.13±0.00f	0.11±0.00e	0.11±0.00e	0.04±0.00b	0.09±0.00d	0.16±0.00g
Wogonoside	0.04±0.00a	0.18±0.00h	0.05±0.00b	0.14±0.00g	7.26±0.28n	0.08±0.00c	0.12±0.00f	0.15±0.01g	0.11±0.00e	1.42±0.05m	0.09±0.00d	0.28±0.01k	0.34±0.00l	0.20±0.00i	0.25±0.01j

6-Methylcoumarin	0.12± 0.00	0.25± 0.00	0.14± 0.00	0.36± 0.01	0.98± 0.02	0.20± 0.00	0.34± 0.01	0.23± 0.00	0.22± 0.00	0.42± 0.01	0.08± 0.00	0.34± 0.00	0.30± 0.00	0.43± 0.01	0.24± 0.01
Quercetin	0.02± 0.00b	0.02± 0.00b	0.01± 0.00a	0.04± 0.00d	0.31± 0.01e	0.01± 0.00a	0.01± 0.00a	0.03± 0.00c	0.02± 0.00b	0.52± 0.01f	0.03± 0.00c	0.04± 0.00d	0.03± 0.00c	0.02± 0.00b	0.04± 0.00d
Scopoletin	26.26± 0.73d	72.60± 1.04k	76.96± 0.98k	47.48± 1.16h	5.88± 0.17a	63.94± 0.55j	53.50± 0.70i	34.90± 1.01f	52.02± 1.37hi	15.68± 0.52b	20.44± 0.77c	43.46± 1.12gh	30.00± 0.27e	42.73± 0.85g	27.91± 0.61de
Camelliaside A	0.08± 0.00e	0.08± 0.00e	0.02± 0.00a	0.03± 0.00b	3.28± 0.12h	0.02± 0.00a	0.04± 0.00c	0.03± 0.00b	0.05± 0.00d	0.15± 0.00g	0.11± 0.00f	0.08± 0.00e	0.05± 0.00d	0.05± 0.00d	0.04± 0.00c
Isoliquiritin	0.72± 0.01g	0.74± 0.02g	0.25± 0.01c	0.23± 0.01c	0.01± 0.00a	0.73± 0.01g	0.50± 0.02e	0.82± 0.02h	0.32± 0.01d	0.11± 0.01b	0.96± 0.02i	1.79± 0.04j	5.13± 0.34l	2.29± 0.18k	0.66± 0.01f
Galangin	0.74± 0.02h	0.26± 0.01d	0.14± 0.00a	0.14± 0.00a	0.26± 0.00d	2.71± 0.05k	0.14± 0.00a	0.37± 0.01f	1.22± 0.03i	0.18± 0.00c	0.33± 0.00e	0.56± 0.02g	2.31± 0.05j	0.41± 0.01f	0.16± 0.00b
Ferulic acid	4.31± 0.07j	0.73± 0.02c	0.59± 0.01b	2.95± 0.08i	0.18± 0.00a	1.31± 0.04e	1.60± 0.03f	1.41± 0.02e	0.92± 0.02d	2.89± 0.07i	2.60± 0.05h	2.38± 0.04h	1.61± 0.05	1.87± 0.02g	2.09± 0.04h
Isoferulic acid	0.74± 0.01j	0.27± 0.01e	0.14± 0.00a	0.15± 0.01ab	0.27± 0.01e	2.71± 0.06m	0.21± 0.01d	0.37± 0.00g	1.22± 0.03k	0.18± 0.00c	0.34± 0.01f	0.56± 0.01i	2.31± 0.05l	0.40± 0.01h	0.16± 0.00b
Demethylnobiletin	0.47± 0.01d	0.96± 0.02h	0.89± 0.01g	0.96± 0.01h	0.02± 0.00a	1.11± 0.03i	0.29± 0.00c	1.21± 0.01j	0.65± 0.02e	1.10± 0.02i	0.13± 0.00b	0.81± 0.01f	0.43± 0.01d	0.80± 0.02f	0.98± 0.02h
Eriodictyol	0.07± 0.00c	0.59± 0.02j	0.03± 0.00a	0.10± 0.00f	0.03± 0.00a	0.05± 0.00b	0.07± 0.00c	0.03± 0.00a	0.09± 0.00e	0.29± 0.01i	0.13± 0.00g	0.29± 0.01i	0.08± 0.00d	0.09± 0.00e	0.22± 0.00h
Kaempferol	0.90± 0.01h	0.34± 0.01c	0.18± 0.00a	0.44± 0.02d	2.10± 0.05k	0.33± 0.01c	0.70± 0.01f	0.84± 0.02h	0.44± 0.01d	1.03± 0.02i	1.21± 0.02j	0.52± 0.01e	0.54± 0.02e	0.29± 0.01b	0.77± 0.02g
Myricetin	0.23± 0.01fg	0.25± 0.00g	0.08± 0.00a	0.32± 0.01i	0.15± 0.00d	0.12± 0.00b	0.19± 0.00e	0.13± 0.00c	0.21± 0.00f	0.56± 0.02k	0.49± 0.01j	0.31± 0.01h	0.13± 0.00c	0.16± 0.01d	0.27± 0.00g
(+)-Catechin	0.06± 0.00d	0.05± 0.00c	0.02± 0.00a	0.05± 0.00c	0.05± 0.00c	0.02± 0.00a	0.02± 0.00a	0.36± 0.01f	0.05± 0.00c	1.08± 0.02g	0.07± 0.00d	0.37± 0.00f	0.04± 0.00b	0.05± 0.00c	0.10± 0.00e

Polydatin	0.06±0.00d	0.23±0.00k	0.03±0.00a	0.17±0.00i	0.39±0.01l	0.10±0.00g	0.21±0.00j	0.08±0.00f	0.11±0.00h	0.21±0.00j	0.10±0.00g	0.07±0.00e	0.04±0.00b	0.04±0.00b	0.05±0.00c
Phloridzin	0.32±0.01f	0.01±0.00a	0.01±0.00a	0.02±0.00b	0.01±0.00a	0.01±0.00a	0.01±0.00a	0.03±0.00c	0.01±0.00a	0.02±0.00b	0.12±0.00e	0.02±0.00b	0.01±0.00a	0.01±0.00a	0.05±0.00d
Esculetin	0.14±0.00i	0.20±0.00j	0.06±0.00c	0.09±0.00f	0.09±0.00f	0.05±0.00b	0.11±0.00g	0.05±0.00b	0.04±0.00a	0.27±0.00k	0.08±0.00e	0.07±0.00d	0.04±0.00a	0.06±0.00c	0.13±0.00h
Angelol B	0.03±0.00c	0.19±0.00f	0.04±0.00d	0.09±0.00e	0.04±0.00d	0.01±0.00a	0.03±0.00c	0.04±0.00d	0.02±0.00b	0.46±0.01g	0.04±0.00d	0.02±0.00b	0.02±0.00b	0.02±0.00b	0.03±0.00c
Casticin	0.26±0.00i	0.13±0.00d	0.05±0.00b	0.27±0.00ij	0.74±0.01l	0.07±0.00c	0.29±0.00j	0.22±0.00h	0.01±0.00a	0.35±0.01k	0.37±0.01k	0.17±0.00g	0.14±0.00e	0.13±0.00d	0.15±0.00f
Trifolirhizin	0.20±0.00g	0.09±0.00e	0.09±0.00e	0.05±0.00b	0.06±0.00c	0.12±0.00f	0.06±0.00c	0.06±0.00c	0.04±0.00a	0.07±0.00d	0.07±0.00d	0.13±0.00f	0.05±0.00b	0.04±0.00a	0.06±0.00c
Pelargonidin chloride	0.06±0.00e	0.07±0.00f	0.04±0.00c	0.03±0.00b	0.03±0.00b	0.05±0.00d	0.02±0.00a	0.08±0.00g	0.16±0.00i	0.04±0.00c	0.05±0.00d	0.12±0.00h	0.18±0.00j	0.08±0.00g	0.07±0.00f
Naringin Dihydrochalcone	0.03±0.00c	0.02±0.00b	0.01±0.00a	0.03±0.00c	0.42±0.00f	0.01±0.00a	0.04±0.00d	0.03±0.00c	0.02±0.00b	0.04±0.00d	0.05±0.00e	0.05±0.00e	0.02±0.00b	0.02±0.00b	0.04±0.00d
Isorhamnetin	0.02±0.00b	0.06±0.00d	0.01±0.00a	0.06±0.00d	0.01±0.00a	0.01±0.00a	0.01±0.00a	0.02±0.00b	0.01±0.00a	0.02±0.00b	0.03±0.00c	0.01±0.00a	0.01±0.00a	0.01±0.00a	0.02±0.00b
Luteolin	0.05±0.00e	0.06±0.00f	0.01±0.00a	0.04±0.00d	0.01±0.00a	0.01±0.00a	0.02±0.00b	0.02±0.00b	0.01±0.00a	0.03±0.00c	0.04±0.00d	0.02±0.00b	0.02±0.00b	0.02±0.00b	0.03±0.00c
Hesperetin 5-O-glucoside	0.04±0.00d	0.02±0.00b	0.03±0.00c	0.04±0.00d	0.01±0.00a	0.02±0.00b	0.02±0.00b	0.08±0.00g	0.06±0.00f	0.08±0.00g	0.05±0.00e	0.04±0.00d	0.05±0.00e	0.05±0.00e	0.06±0.00f
Naringenin	0.05±0.00e	0.02±0.00b	0.01±0.00a	0.08±0.00h	0.06±0.00f	0.02±0.00b	0.05±0.00e	0.06±0.00f	0.04±0.00d	0.07±0.00g	0.07±0.00g	0.07±0.00g	0.05±0.00e	0.03±0.00c	0.07±0.00g
Rhapontin	0.03±0.00c	0.01±0.00a	0.01±0.00a	0.02±0.00b	0.05±0.00e	0.01±0.00a	0.01±0.00a	0.02±0.00b	0.02±0.00b	0.03±0.00c	0.03±0.00c	0.04±0.00d	0.02±0.00b	0.03±0.00c	0.03±0.00c

Orientin	0.01± 0.00a	0.01± 0.00a	0.01± 0.00a	0.01± 0.00a	0.06± 0.00b	0.01± 0.00a	0.01± 0.00a	0.01± 0.00a	0.01± 0.00a	0.01± 0.00a	0.01± 0.00a	0.01± 0.00a	0.01± 0.00a	0.01± 0.00a	0.01± 0.00a
Delphinidin chloride	0.01± 0.00a	0	0	0.01± 0.00a	0.01± 0.00a	0	0.01± 0.00a	0.01± 0.00a	0	0.01± 0.00a	0.01± 0.00a	0.01± 0.00a	0.02± 0.00b	0.01± 0.00a	0.01± 0.00a
Schisantherin A	0.01± 0.00a	0.01± 0.00a	0	0.01± 0.00a	0.02± 0.00b	0	0	0.01± 0.00a	0.01± 0.00a	0.01± 0.00a	0.01± 0.00a	0.01± 0.00a	0.01± 0.00a	0.01± 0.00a	0.01± 0.00a
Tectorigenin	3.37± 0.05g	1.74± 0.02cd	0.75± 0.02a	1.97± 0.05e	2.68± 0.07f	1.34± 0.01b	5.40± 0.04j	2.72± 0.10f	1.38± 0.06b	5.04± 0.06i	4.60± 0.03h	1.87± 0.02de	1.82± 0.02d	1.61± 0.03c	2.66± 0.06f
Apigenin	0.09± 0.00e	0.04± 0.00b	0.03± 0.00a	0.09± 0.00e	0.17± 0.00j	0.04± 0.00b	0.10± 0.00f	0.12± 0.00g	0.06± 0.00c	0.15± 0.00i	0.14± 0.00h	0.07± 0.00d	0.07± 0.00d	0.07± 0.00d	0.12± 0.00g
Schisandrin A	0.94± 0.01i	0.19± 0.00b	0.19± 0.00b	0.72± 0.01h	0.10± 0.00a	0.28± 0.00d	0.49± 0.01e	0.46± 0.00e	0.29± 0.00d	0.66± 0.02gh	0.60± 0.01g	0.73± 0.01h	0.54± 0.00f	0.23± 0.00c	0.68± 0.02h
Isoliquiritigenin	0.04± 0.00d	0.03± 0.00c	0.01± 0.00a	0.15± 0.00h	0.49± 0.01j	0.02± 0.00b	0.03± 0.00c	0.05± 0.00e	0.03± 0.00c	0.29± 0.00i	0.05± 0.00e	0.52± 0.01j	0.08± 0.00g	0.04± 0.00d	0.06± 0.00f
Rhapontigenin	1.07± 0.03k	0.52± 0.01h	0.17± 0.00b	0.58± 0.01i	0.01± 0.00a	0.24± 0.00c	0.35± 0.01e	0.31± 0.00d	0.39± 0.01f	0.46± 0.01g	0.82± 0.02j	1.47± 0.03l	0.31± 0.00d	0.50± 0.01gh	0.51± 0.01h
Epicatechin	2.05± 0.06j	0.31± 0.00b	0.12± 0.00a	2.21± 0.04j	0.43± 0.01d	0.50± 0.01e	3.34± 0.07k	0.55± 0.01f	1.13± 0.03h	1.21± 0.02h	5.49± 0.11l	0.83± 0.02g	0.40± 0.01d	0.35± 0.00c	1.78± 0.03i
Chrysin	0.09± 0.00e	0.57± 0.01h	0.01± 0.00a	0.08± 0.00d	0.11± 0.00f	0.09± 0.00e	0.21± 0.00g	0.03± 0.00c	0.02± 0.00b	0.03± 0.00c	0.60± 0.01h	0.02± 0.00b	0.02± 0.00b	0.02± 0.00b	0.55± 0.02h
Schisanhenol	1.28± 0.03j	0.70± 0.00f	0.77± 0.01gh	0.99± 0.02i	0.04± 0.00a	0.81± 0.02h	0.29± 0.00c	0.62± 0.01e	0.23± 0.00b	0.50± 0.02d	0.74± 0.01fg	0.60± 0.01e	0.63± 0.02e	0.52± 0.01d	2.05± 0.03k
Schizandrol A	0.04± 0.00b	0.05± 0.00c	0.03± 0.00a	0.04± 0.00b	6.96± 0.11j	0.05± 0.00c	0.08± 0.00e	0.08± 0.00e	0.07± 0.00d	0.15± 0.00i	0.04± 0.00b	0.10± 0.00g	0.09± 0.00f	0.07± 0.00d	0.11± 0.00h
Procyanidin B2	1.76± 0.02d	0.27± 0.00b	1.15± 0.02c	2.63± 0.05g	0.15± 0.00a	2.29± 0.04f	0.27± 0.00b	7.06± 0.16j	5.62± 0.08i	1.98± 0.04e	4.64± 0.11h	1.91± 0.06e	2.39± 0.07fg	2.00± 0.03e	12.62± 0.42k

Schisandrin C	0.09± 0.00f	0.02± 0.00a	0.04± 0.00c	0.05± 0.00d	0.05± 0.00d	0.04± 0.00c	0.03± 0.00b	0.12± 0.00h	0.07± 0.00e	0.73± 0.01l	0.07± 0.00e	0.30± 0.00k	0.24± 0.00j	0.19± 0.00i	0.11± 0.00g
Cyanidin 3-rutinoside	1.70± 0.03j	0.07± 0.00a	0.22± 0.00g	0.16± 0.00f	0.13± 0.00d	0.24± 0.00h	0.12± 0.00c	0.13± 0.00d	0.08± 0.00b	0.14± 0.00e	0.24± 0.00h	0.26± 0.00i	0.27± 0.01i	0.27± 0.00i	0.20± 0.00g
Coumarin 151	0.06± 0.00d	0.02± 0.00a	0.02± 0.00a	0.06± 0.00d	0.12± 0.00g	0.02± 0.00a	0.06± 0.00d	0.07± 0.00e	0.03± 0.00b	0.07± 0.00e	0.09± 0.00f	0.04± 0.00c	0.04± 0.00c	0.04± 0.00c	0.09± 0.00f

JSXZ, Jin Si Xiao Zao; NHDZ, Nei Huang Da Zao; SZ, Suan Zao; HMDZ, Ha Mi Da Zao; HZ, Hui Zao; LZYZ, Lin Ze Yu Zao; JSBZ, Ji Shan Ban Zao; ZHDZ, Zan Huang Da Zao; TZ, Tan Zao; XSHZ, Xi Sha Hong Zao; RQHZ, Ruo Qiang Hui Zao; JCJZ, Jiao Cheng Jun Zao; HTDZ, He Tian Da Zao; HPZ, Hu Ping Zao; NYDZ, Ning Yang Da Zao. Mean values with different lower-case letters in the same column correspond to significant differences at $p < 0.05$. Data are represented as the mean ± SD (standard deviation).
